# Follicular hyperandrogenism and insulin resistance in polycystic ovary syndrome patients with normal circulating testosterone levels

**DOI:** 10.7555/JBR.32.20170136

**Published:** 2018-03-12

**Authors:** Andi Li, Lu Zhang, Jiajia Jiang, Nan Yang, Ying Liu, Lingbo Cai, Yugui Cui, Feiyang Diao, Xiao Han, Jiayin Liu, Yujie Sun

**Affiliations:** 1. Key Laboratory of Human Functional Genomics of Jiangsu Province, Nanjing Medical University, Nanjing, Jiangsu 211166, China; 2. State Key Laboratory of Reproductive Medicine, Clinical Center of Reproductive Medicine, the First Affiliated Hospital of Nanjing Medical University, Nanjing, Jiangsu 210029, China; 3. Department of Cell Biology, Nanjing Medical University, Nanjing, Jiangsu 211166, China.

**Keywords:** polycystic ovary syndrome, hyperandrogenism, insulin resistance, follicular fluid

## Abstract

Polycystic ovary syndrome (PCOS) is a common reproductive disease with high heterogeneity. The role of excess androgen in PCOS etiology remains disputed, since around 20%–50% of PCOS women do not display hyperandrogenemia. The microenvironment of the ovary critically influences follicular development. In the present study, we assessed the role of androgen in PCOS by investigating whether excessive follicular fluid androgen was present in PCOS patients with normal serum androgen levels and influenced by follicular fluid insulin resistance (IR). Follicular fluid samples of 105 women with PCOS and 105 controls were collected. Levels of steroid hormones, glucose and insulin in the follicular fluid were examined and compared with data from serum biochemistry tests. We found that 64.9% (63/97) of PCOS patients with normal serum androgen levels displayed abnormally high follicular fluid androgen level. The follicular fluid androgen level was positively correlated with follicular fluid IR within a certain range and follicular fluid estrogen-to-testosterone (E2/T) ratio was significantly reduced in these patients. These results indicated that there existed a subgroup of PCOS patients who displayed excessive follicular fluid androgen and IR despite their normal circulating testosterone (T) levels. Our study highlights the importance of ovary hyperandrogenism and IR in the etiology of PCOS.

## Introduction

Polycystic ovary syndrome (PCOS) is a common endocrine disease as well as a metabolic disorder with heterogeneous presentations^[1]^. It affects 6%–18% of women of reproductive age^[2^–^4]^. It is associated not only with reproductive and obstetric problems including hyperandrogenism (HA), menstrual dysfunction, infertility and pregnancy complications, but also with metabolic and long-term health risks such as dyslipidemia, insulin resistance (IR), elevated risk of impaired glucose tolerance, type 2 diabetes and metabolic syndrome^[5^–^6]^. For women with PCOS who suffer from non-ovulation, induction of ovulation with pharmacological agents constitutes the first line treatment, but the curative effects vary greatly because of high heterogeneity of the disease. Nowadays, our knowledge on the etiology and development of PCOS is still very limited.

PCOS diagnosis is mainly based on the presence of at least two of Rotterdam criteria (chronic anovulation, HA, and polycystic ovary) established in 2003. In 2006, the Androgen Excess & PCOS Society recommended androgen excess as a central feature of the disease and that PCOS should be defined by the presence of HA (clinical and/or biochemical) in combination with ovarian dysfunction (anovulation and/or polycystic ovary), emphasizing HA as a biochemical hallmark of PCOS. However, the prevalence of PCOS women with HA varies in different ethnic populations. For example, the elevated circulating androgen levels are observed in nearly 80% American women^[7]^ and over 50% European^[8]^ women with PCOS, while the prevalence is around 24% in mainland China^[3]^, which is much lower than that in Europe or America.

The microenvironment of the ovary has a direct effect on follicular development. It has been reported that the follicular fluid had a higher testosterone (T) level in patients with PCOS than in the control group^[9]^. At present, it is unclear whether excessive follicular fluid androgen can be reflected by circulating androgen level reliably and to what extent excessive follicular fluid androgen impacts on PCOS etiology and development. Therefore, identifying the relationship between circulating and follicular fluid androgen levels is significant for diagnosis and treatment of PCOS.

IR, another central component of PCOS, has also been observed in 20%–40% of women with PCOS^[6]^. Hyperinsulinemia was reported to produce a hyperandrogenic state by acting as a co-gonadotropin with luteinizing hormone (LH). Lowering insulin with metformin decreases circulating androgen level^[10]^. At present, it is still unclear whether insulin level in the follicular fluid influences follicular local androgen level. Determination of the relationship between the insulin and androgen levels in the follicular local environment will help to understand the roles of both follicular local HA and IR in the process of ovarian dysfunction.

Our study was designed to determine whether follicular fluid androgen level was consistent with the circulating androgen level in PCOS patients and the controls and whether the follicular androgen level was related to ovary IR.

## Patients and methods

### Patients

One hundred and five patients diagnosed with PCOS at the Reproductive Center of the First Affiliated Hospital of Nanjing Medical University who were admitted between April, 2012 and March, 2015 were enrolled in this study. At the same time, 105 infertile women of childbearing age with tubal blockage or whose husbands had azoospermatism were chosen as the control group. PCOS patients were diagnosed according to the 2003 Rotterdam criteria as follows: (1) ovulatory dysfunction, oligomenorrhea, or amenorrhea, eight or fewer menstrual cycles in 1 year or menstrual cycles>35 days in length; (2) polycystic ovaries, more than 12 follicles with the size of 2-9 mm in each ovary and/or unilateral ovarian volume of >10 mL on ultrasound; (3) HA, including clinical manifestations of hirsutism, severe acne, and exclusion of other etiologies (e.g. congenital adrenal hyperplasia, androgen secreting tumors, or Cushing’s syndrome). Patients must meet at least two of the above criteria and have no other diseases that can result in similar symptoms of polycystic ovaries and HA. Clinical features, including menstrual cycle, anthropometric variables, as well as endocrine and biochemical parameters were recorded.

This study was approved by the Ethics Committees of Nanjing Medical University. Patient consent was not required because of the retrospective nature of this study.

### Collection of serum and follicle fluid samples

Blood samples were collected by the Reproductive Center of the First Affiliated Hospital of Nanjing Medical University. The samples were collected on days 2–3 of the menstrual cycle or during amenorrhea in PCOS patients. The blood samples containing 10% Na_2_ethylenediaminetetraacetic acid were centrifuged at 1,300 *g* for 20 minutes at 4 °C, and the plasma was stored at -80 °C for analysis. Follicular fluid samples were obtained during the oocyte retrieval operation by aspiration from the first-punctured follicle with 18 to 25 mm in diameter in each subject. The samples were then centrifuged at 800 *g* for 10 minutes at 4 °C, and the supernatants were stored at -80 °C for further examination.

### Measurement of steroid hormones

Data of circulating levels of steroid hormones, including T, E2, follicle stimulating hormone (FSH) and LH, were obtained from the Reproductive Center of the First Affiliated Hospital of Nanjing Medical University^[11]^. T and E2 in follicular fluid were measured using radio immunoassay (RIA) kits according to the manufacturer’s instructions (Beijing North Biotechnology Research Institute). The intra- and inter-assay coefficients of variation for the total T assay were<10% and<15% at 0.1–20.0 ng/mL respectively (the sensitivity was 0.02 ng/mL). The intra- and inter-assay coefficients of variation for the E2 assay were<10% and<15% at 5–4,000 pg/mL, respectively (the sensitivity was<2 pg/mL). Based on the hospital definition, total T ≥ 2.6 nmol/L (0.7 ng/mL) was defined as biochemical HA. Since no standard of follicular fluid HA was given, follicular fluid androgen excess was defined as total T (ng/mL) higher than the upper limit of the 95% CI of the control group (3.298 ng/mL).

### Analysis of insulin resistance in the follicular fluid

Follicular fluid insulin concentration was measured by RIA kits according to the manufacturer’s instructions (Beijing North Biotechnology Research Institute). The intra- and inter-assay coefficients of variation for the insulin assay were, respectively, 3.4% and 3.6% at 11 mIU/L, and 4.0% and 2.6% at 36 mIU/L. The follicular fluid glucose was measured by Roche Cobas c501 automatic biochemical analyzer based on spectrophotometry (the sensitivity was 0.11 mmol/L). Follicular fluid HOMA-IR, calculated as follicular fluid glucose × follicular fluid insulin/22.5, was used to determine the IR level in follicular fluid.

### Statistical analysis

Data were analyzed using the Statistical Package for Social Sciences (SPSS 21.0 for Windows). Distributions were compared using Student’s *t*-test or Mann-Whitney *U* as appropriate. Chi-square analysis or Fisher exact test was used to compare the prevalence of obesity and HA between the PCOS and control groups. PCOS patients were divided into two groups using 95% confidence-interval (CI) upper limit of the control group as a cutoff value. Group 1 was defined to have normal follicular fluid T level and Group 2 displayed abnormally high follicular fluid T level. Pearson’s correlation coefficient was applied to investigate the correlation between clinical and hormonal levels and metabolic levels in serum and follicular fluid. *P*<0.05 was considered statistically significant.

## Results

### The baseline characteristics of the study patients 

A total of 210 Chinese Han females, 105 women with PCOS diagnosed based on the 2003 Rotterdam criteria and 105 control women were randomly chosen. The baseline characteristics of the 105 PCOS patients are shown in ***Table 1***. Among 105 women with PCOS, 98 (93.3%) displayed typical polycystic ovaries, which is consistent with the other reported studies in Chinese (94.2%)^[3]^ and European populations (90%–95%)^[12]^.There was no significant difference in height, weight and body mass index (BMI) (*P* = 0.56, 0.21, and 0.1, respectively) between the PCOS group and the control group (***Table 1***). The average age of women with PCOS was 28.88±3.93, one year older than the women in the control group (27.74±3.89 years) (*P* = 0.04). Nineteen out of 105 patients (18%) were overweight (BMI ≥ 25), which was lower than that in the reported data^[13]^.

**Tab.1:** The baseline characteristics of women with PCOS and the control group

Characteristics	Controls	PCOS	*P* value
N	105	105	
Age (years)	27.74±3.89	28.88±3.93	0.04^a^
Height (cm)	160.7±4.89	160.29±5.29	0.56^a^
Weight (kg)	56.78±7.01	58.06±7.75	0.21^a^
BMI (kg/m^2^)	21.98±2.52	22.59±2.7	0.10^a^
Polycystic ovaries (*n*)	not done	98	none
Obesity(BMI≥25 kg/m^2^) [*n*(%)]	9 (8.57)	19 (18.10)	0.04^b^
HA [*n*(%)]	5 (4.76)	8 (7.62)	0.39^b^

values are presented as mean±SD; Univariate analysis: qualitative variables were analyzed through χ^2^ test or Fisher exact test; BMI: body mass index; HA: hyperandrogenemia. ^a^ Student’s *t* test; ^b^ Chi-square test %.

### T levels and the E2-to-T ratio in follicular fluids of PCOS women

**Fig.1:**
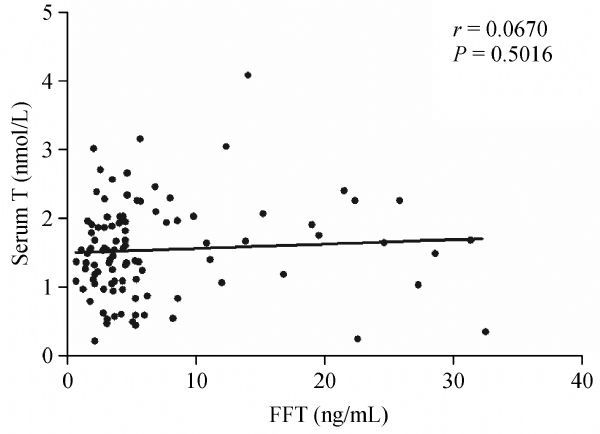
The follicular fluid testosterone (T) level is not correlated with serum T level in PCOS patients. There is no correlation between follicular fluid and serum T in women with PCOS, indicating the follicular fluid T level was independent of the circulating T level.

Hyperandrogenism is thought to be a hallmark of PCOS. But in our present study, only 7.6% (8/105) of women with PCOS displayed biochemical HA (T>2.6 nmol/L). The average circulating T level was 2.5 (1.06–1.94) nmol/L in women with PCOS, which was not significantly different from 2.11 (0.9–1.63) nmol/L in the control group (*P* = 0.67). Likewise, the mean circulating E2 level was (128.58±76.85) pg/mL in the PCOS group which had no significant difference from (140.43±81.06) pg/mL in the control group (*P* = 0.28). Subsequently, we studied whether the T level in the follicular fluid was different between PCOS patients and the controls. As indicated in*** Table 2***, the follicular fluid T level of women with PCOS was 6.77 (2.41–5.83) ng/mL, which was significantly higher than 2.87 (1.61–3.03) ng/mL in the control group (*P *<0.01). However, no positive correlation between circulating and follicular T levels was observed (***Fig. 1***; *r* = 0.0670, *P* = 0.5016). No significant difference was found in follicular fluid T levels between PCOS patients with or without high circulating androgen levels (*P* = 0.68). These data suggested that the follicular fluid T level in PCOS patients could be abnormally increased independent of the circulating T level, implying that increased follicular fluid T may be an independent factor of PCOS.

Interestingly, the follicular fluid estrogen level was not increased in PCOS patients (*P* = 0.76), although the T level was increased in the local environment. However, follicular fluid E2/T was significantly decreased in the PCOS group (5.42), when compared with the control group (10.45, *P*<0.01). This meant that the transformation of T to E2 might be impaired in PCOS patients with excessive follicular fluid androgen.

### Follicular fluid T level was positively correlated with follicular fluid HOMA-IR values within a certain range

**Fig.2:**
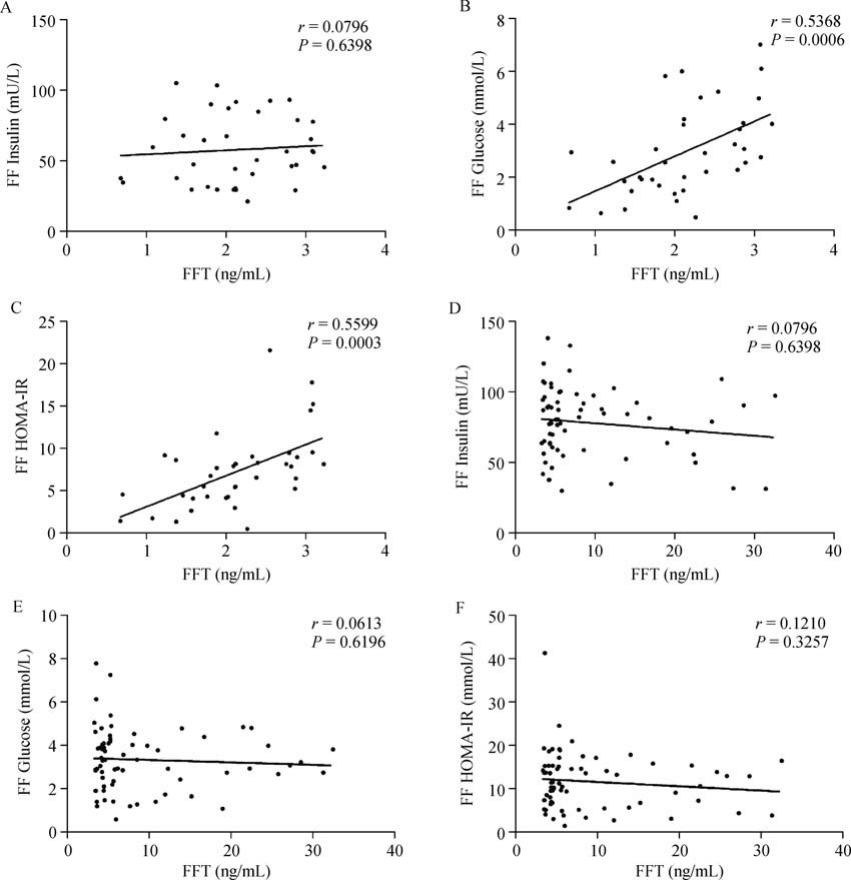
Correlation of follicular fluid T and follicular fluid insulin resistance (IR) in the PCOS patients. PCOS patients were divided into two groups as based on the follicular fluid T level: Group 1 with normal follicular fluid T level (A-C) group 2 with abnormally increased follicular fluid T level (D-F). In Group 1, follicular fluid T was positively correlated with glucose and HOMA-IR (B and C), P < 0.05, although no significant correlation of follicular fluid T with follicular fluid insulin was detected (A). In Group 2, follicular fluid T was not significantly correlated with insulin (D), glucose (E) and HOMA-IR (F) within the follicular fluid, suggesting that in a certain range follicular fluid T level might be influenced by follicular fluid insulin level.

IR has been found to have promoting effects on HA^[14]^. We determined whether follicular fluid IR contributed to excessive follicular fluid androgen by testing follicular fluid insulin and glucose levels with RIA. Our data showed that the glucose level in the follicular fluid was significantly higher in PCOS women when compared with the control group (*P* = 0.02), but no significant difference of the insulin level was detected (***Table 2***). The follicular fluid HOMA-IR value was 10.05±6.17 in the PCOS group, and was significantly higher than 7.66±4.33 in the control group (*P*<0.01). In Group 1 follicular fluid T was found to be positively correlated with follicular fluid glucose level (***Fig. 2B***; *r* = 0.5368, *P* = 0.0006, 95% CI: 0.2575-0.7333) and HOMA-IR (***Fig. 2C***; *r* = 0.5599, *P*= 0.0003; 95% CI: 0.2881-0.7482), although it was not significantly correlated with follicular fluid insulin (***Fig. 2A***; *r* = 0.0796, *P* = 0.6398). Paradoxically, in Group 2 follicular fluid T showed no significant correlation with insulin (***Fig. 2D***; *r *= -0.1364, *P* = 0.2426), glucose (***Fig. 2E***; *r* = -0.0613, *P* = 0.6196) and HOMA-IR (***Fig. 2F***; *r* = -0.1210, *P* = 0.3257) in the follicular fluid. These data suggested that ovary IR might be a promoting factor of high ovary T level in a subgroup of PCOS patients.

**Tab.2:** Hormonal and metabolic levels in PCOS patients and controls

	Controls (*N*=105)	PCOS (*N*=105)	*P* value
Serum			
FSH (mU/L)	7.05±1.79	6.17±1.54	<0.01
LH (mU/L)	4.42±1.78	7.18±5.23	<0.01
LH/FSH ratio	0.66±0.32	1.23±1.06	<0.01
E2 (pg/mL)	128.58±76.85	140.43±81.06	0.28
T (nmol/L)	2.11 (0.9-1.63) ^#^	2.5 (1.06-1.94)^ #^	0.67
E2/T ratio	138.21 (61.86-138.99)^ #^	109.14 (53.40-120.16)^ #^	0.18
Follicular fluid			
FF T (ng/mL)	2.87(1.61-3.03)^ #^	6.77 (2.41-5.83)^ #^	<0.01
FF E2 (ng/mL)	17.88±9.07	18.28±10.15	0.76
FF E2/T ratio	10.45 (3.74-10.64)^ #^	5.42 (1.79-6.08)^ #^	<0.01
FF Glu (mmol/L)	3.2±1.29	3.49±1.40	0.02
FF Ins (mU/L)	66.76±27.79	70.63±25.87	0.30
FF HOMA-IR	7.66±4.33	10.05±6.17	<0.01

values are presented as mean±SD; ^#^ mean (IQR). The Mann-Whitney *U*-test was carried out. FSH: follicle-stimulating hormone; LH: luteinizing hormone; T: testosterone; E2: estrogen; FF: follicular fluid; Glu: glucose; Ins: insulin; HOMA-IR: insulin resistance level.

## Discussion

In this study, we found that follicular fluid androgen level could be significantly increased in women with PCOS even though these patients displayed normal circulating androgen level. Such ovary HA was partially positively correlated with the local IR level in the ovary. These observations have important implications for improvement of PCOS diagnosis and treatment as well as for understanding of PCOS etiology.

Although HA is thought to be responsible for oligo-ovulation and follicular atresia, varied prevalence of PCOS women with HA has been reported and the role of androgens in PCOS etiology is still an open question. Furthermore, little is known about the relationship between circulating and ovary HA in PCOS patients, though they could occur together in patients with PCOS^[15–16]^.

In the present study, the frequency of PCOS patients with biochemical androgen excess was only 7.62% (8/105), lower than expected. In contrast, the significant higher frequency (64.8%, 68/105) of ovary androgen excess was detected in the PCOS patients. Ovary androgen excess was not always in parallel with that in circulating serum. PCOS women who displayed normal circulating androgen levels had great possibility to have excessive ovary androgen. These observations, on one hand, supported the critical role of abnormal androgen metabolism in the etiology of PCOS. On the other hand, our study implied a non-negligible limitation of using excessive circulating androgen as diagnostic criteria for PCOS. Ovary HA could be a hidden and more direct factor of ovarian malfunctions. These findings had potential clinical and therapeutic implications and suggested that abnormal ovary androgen metabolism should be carefully considered in diagnosis and treatment of PCOS.

BMI has been confirmed to be positively related to circulating androgen excess. The low percentage of PCOS patients with circulating biochemical HA observed in this study might be partly explained by the low rate of overweight PCOS patients we collected, since losing weight, rather than IVF, was a preferred choice for overweight PCOS patients at our Center. The independent increase of follicular fluid T in PCOS patients and its diagnostic value deserved further explorations in large samples.

HA and IR are considered to be strongly associated with PCOS^[17^–^20]^. The HOMA-IR (plasma glucose × plasma insulin/22.5) is a well-defined criterion for evaluation of IR, which is correlated with the gold standard clamp test for IR. Since there is still no standard to measure IR level in the follicular fluid, we used HOMA-IR to estimate IR in the ovary in this study. We found that the level of follicular fluid HOMA-IR was significantly higher in women with PCOS than in the control group. Chi-square analysis showed that follicular fluid IR was positively correlated with follicular fluid HA either in the PCOS group or the control group. These data strongly suggest that ovary IR and HA may intrinsically interact with each other. Little is known about the intra-ovary interaction between T and IR. Few reports available are conflicting. For example, T treatment was reported to down-regulate Glut4 expression and downstream insulin signaling, leading to IR in endometrial cells^[21]^, while other reports showed HA had no significant effect on insulin signaling in granulosa cells^[22]^. On the other hand, insulin was reported to stimulate androgen production by enhancing the expression and activation of P450c17 in theca cells *in vitro*^[23^–^25]^*.* The follicular fluid IR might prevent follicular fluid T from converting to E2, since the E2/T level was significantly lower in the follicular fluid of PCOS patients. Determination of intro-ovary CYP19a1 activity and expression of PCOS patients with follicular fluid HA and follicular fluid IR will provide useful clues. At present, we do not know how excessive follicular fluid androgen could exist in PCOS patients with normal androgen level in serum. We proposed that follicular fluid IR may partly contribute to the local high androgen, since follicular fluid T level was positively correlated with follicular fluid HOMA-IR values. Follicular fluid androgen excess and IR might disturb local hormone metabolism, thus contributing to the pathogenesis of PCOS.

Another interesting question raised by this study is whether ovary IR could occur independent of circulating IR level as in the case of HA. Unfortunately, the data of circulating insulin level of the patients examined in this study were not available. Further investigation is required.

One limitation of the present study is the small sample size of the PCOS patients with high circulating androgen levels. Further study with a larger patient population is required to determine whether follicular fluid T levels are even higher in PCOS patients with high circulating androgen levels than those without.

In conclusion, our present study revealed that a large proportion of PCOS patients with normal circulating androgen displayed follicular fluid HA. Follicular fluid T level was positively correlated with follicular fluid IR in a certain range. Our study highlights the potential roles of ovary androgen excess and IR in PCOS etiology and has significant implications for PCOS diagnosis and treatment.
